# Fast Pencil Beam Dose Calculation for Proton Therapy Using a Double-Gaussian Beam Model

**DOI:** 10.3389/fonc.2015.00281

**Published:** 2015-12-18

**Authors:** Joakim da Silva, Richard Ansorge, Rajesh Jena

**Affiliations:** ^1^Cavendish Laboratory, Department of Physics, University of Cambridge, Cambridge, UK; ^2^Department of Oncology, University of Cambridge, Cambridge, UK

**Keywords:** pencil beam, proton therapy, dose calculation, double Gaussian, graphics processing unit, adaptive radiotherapy

## Abstract

The highly conformal dose distributions produced by scanned proton pencil beams (PBs) are more sensitive to motion and anatomical changes than those produced by conventional radiotherapy. The ability to calculate the dose in real-time as it is being delivered would enable, for example, online dose monitoring, and is therefore highly desirable. We have previously described an implementation of a PB algorithm running on graphics processing units (GPUs) intended specifically for online dose calculation. Here, we present an extension to the dose calculation engine employing a double-Gaussian beam model to better account for the low-dose halo. To the best of our knowledge, it is the first such PB algorithm for proton therapy running on a GPU. We employ two different parameterizations for the halo dose, one describing the distribution of secondary particles from nuclear interactions found in the literature and one relying on directly fitting the model to Monte Carlo simulations of PBs in water. Despite the large width of the halo contribution, we show how in either case the second Gaussian can be included while prolonging the calculation of the investigated plans by no more than 16%, or the calculation of the most time-consuming energy layers by about 25%. Furthermore, the calculation time is relatively unaffected by the parameterization used, which suggests that these results should hold also for different systems. Finally, since the implementation is based on an algorithm employed by a commercial treatment planning system, it is expected that with adequate tuning, it should be able to reproduce the halo dose from a general beam line with sufficient accuracy.

## Introduction

Fast dose calculation finds use in a variety of radiotherapy applications and is an active area of research (1). Due to the high level of dose conformity, the small number of treatment fields, and the sensitivity to material changes in the beam path, adaptive treatment techniques relying on fast, repeated dose calculation are of particular interest in proton therapy. A considerable amount of work has therefore gone into using graphics processing units (GPUs) to speed up proton therapy Monte Carlo (MC) dose calculation in order to allow daily dose recalculation (2–6). However, more advanced adaptive techniques, such as real-time dose monitoring, would involve calculating the dose online as it is being delivered. For a treatment employing pencil beam (PB) scanning, this would require the calculation time of individual energy layers to be short in comparison with the time between energy layers or the length of a typical motion phase. For such applications, GPU MC dose calculation on a single workstation remains too slow by at best one, and generally two or more, orders of magnitude. In a previous paper, we therefore presented a GPU implementation of the widely used PB algorithm, especially developed for use in online calculation (7). The presented dose calculation engine was capable of calculating a two-field, base-of-skull test case in 0.22 s, with individual energy layers of the same case taking 6.4 ms or less to calculate. The short calculation times were largely attributed to the efficient GPU implementation of the computationally expensive kernel superposition (KS) step of the algorithm (8). Although the accuracy of the calculation in the high- and medium-dose regions was seen to be high, with γ-index passing rates matching those of a PB algorithm in clinical use, the implementation used a single-Gaussian kernel to describe the lateral dose profiles of PBs. It is well-known that such a beam model cannot accurately predict the low-dose halo made up of particles traveling at large angles with the beam direction, originating from nuclear interactions, inhomogeneous scattering in the nozzle, or large-angle Rutherford scattering. Despite the low halo dose, their large widths mean that the halos from a number of PBs may overlap to produce a noticeable impact on the overall dose distribution. Modeling of the halo dose is therefore necessary to predict the field-size dependence of the central dose in energy layers. In addition, the halos are responsible for the low-dose region further away from the target, which might be of interest when trying to predict the risk of developing side effects or secondary tumors.

Although PB algorithms for proton therapy have traditionally employed the single-Gaussian beam model, the above reasons have led to modern treatment planning systems (TPSs) almost exclusively making use of more complex models. Generally, these add one or more additional terms for modeling the halo dose to the Gaussian kernel describing the contribution from primary particles. A common method is to use a second, wider Gaussian to describe the halo, an approach first suggested for dose calculation in electron therapy (9). The potential to use the same approach in proton therapy was later pointed out in a paper describing the implementation of a commercial TPS (10). Pedroni et al. presented the first implementation of a double-Gaussian beam model for scanned PBs, using a parameterization based on measurements of the increase in central dose in square fields of increasing side length (11). Later the same year, Soukup et al. presented a different implementation, where the parameterization was instead based on MC simulations of nuclear interactions in water, which was adopted in the commercial TPS XiO Proton (Elekta AB, Stockholm, Sweden) (12). Since then a range of parameterizations, based on measurements, MC simulations, and analytical calculations, have been presented (13–17). Furthermore, several extensions to the double-Gaussian model have been suggested, including using a double-Gaussian model also for the PB shape in air, adding a third Gaussian to the beam model, and adding different non-Gaussian functions to the kernel (18–24).

From the above discussion, it is clear that to be fully comparable to a modern TPS, a calculation engine for online dose monitoring must include a model for the halo dose. Starting with an existing implementation, a double-Gaussian beam model could easily be implemented by simply rerunning the calculation a second time for the halo contribution. The difficulty in a fast implementation, however, stems from the width of the halo: for a double-Gaussian beam model, the width of the halo contribution is expected to be about two to three times larger than that of the primary contribution. The most time-consuming step of the PB algorithm is the KS, where the computational PBs (CPBs) – the computational elements of the PB algorithm obtained from the sub-PB splitting of physical PBs (7) – are widened perpendicular to the beam direction through a superposition of the kernels describing their lateral shape. The number of voxels reached by the kernel at a given depth, and thereby the calculation time of the KS step, is proportional to the square of the CPB width and inversely proportional to the square of the CPB spacing. Therefore, the calculation of a two to three times wider halo dose is expected to take four to nine times longer than that of the primary contribution, threatening to make the calculation time prohibitively long for real-time applications. Here, we describe the integration of a double-Gaussian beam model, based on the algorithm by Soukup et al. (12), into our previously presented GPU dose calculation engine, which aims to reduce the calculation time of the halo dose. It does so in part by employing the method described in Ref. (12), where, in the calculation of the halo dose, a single “nuclear” PB, henceforth referred to as halo PB (HPB), is assigned to each physical PB. Assuming 3 mm PB spacing and 1 mm CPB spacing for the primary contribution, this lack of sub-PB splitting reduces the number of HPBs, and thus their computational load, by a factor of nine. In addition, we employ a separate beam’s-eye-view (BEV) coordinate system for the halo dose. This is defined in the same way as the CPB coordinate system described in our previous implementation (7) but is based on the HPB grid spacing. Using the same assumption as above, this effectively reduces the resolution of the halo calculation by a factor of three, and thereby the computational load of the KS by another factor of nine. Thus, using this approach, the time required by the KS step for the HPBs is expected to be not more than 11% of that for the primary CPBs. Although the main focus of the work was the implementation of the double-Gaussian beam model and its performance, it also includes a comparison of two parameterization approaches for the double-Gaussian beam model, so that their effect on the calculation time could be investigated.

## Materials and Methods

### Algorithm

The PB implementation presented in this paper assumes that the dose *D* to a point (*x*, *y*, *z*) can be described by a double-Gaussian beam model according to

(1) D(x, y, z)=∑i∈CPB(1−u(Ei, zWE,i))×Ni×IIDD(Ei,zWE,i)×G(x−xi,y−yi,σCPB,i) +∑j∈HPBu(Ej,zWE,j)×Nj×IIDD(Ej,zWE,j)×G(x−xj,y−yj,σHPB,j)

The first sum on the right-hand side of Eq. 1 represents the dose from primary particles, which is calculated according to the original implementation using the single-Gaussian beam model presented elsewhere (7). Consequently, the index of summation, *i*, runs over the CPBs resulting from the sub-PB splitting of the physical PBs, and each factor inside the summation (except for the first one) is further identical to what was previously presented. Specifically, *N*_i_ is the CPB weight, *E*_i_ is the initial beam energy, *z*_WE,i_ is the water-equivalent path length (WEPL), *I*_IDD_ is the integral depth dose (IDD), *G* is the Gaussian function, and σ_CPB,i_ is the SD of the primary Gaussian, henceforth referred to as the CPB width. In Eq. 1, *u* (*E*_i_, *z*_WE,i_) ∈ [0,1] is the halo fraction, defined as the share of the integral dose that is deposited by the halo, which is given by the halo dose parameterization. Consequently, the factor [1 − *u*(*E*_i_, *z*_WE,i_)] gives the fraction of the integral dose at a given WEPL that is deposited by primary particles. It should be noted that, although the CPB widths are calculated as described in the original publication, the values of the parameters *E*_S_ and δ, which enter the width calculation as free parameters in the implementation, have to be adjusted in the double-Gaussian beam model. This is because the values (14.1 MeV and 0.21 mm, respectively) obtained for the single-Gaussian beam model were based on how well the shape of the calculated PBs reproduced the total dose distributions, including contributions from the low-dose halo, from individual PBs as obtained by MC simulations. When employing the double-Gaussian model, these should instead be determined by a fit to the primary contribution alone. Therefore, the contribution from the halo should be subtracted from the total dose before finding the best values of *E*_S_ and δ, and these must therefore be determined separately for each halo dose parameterization.

The dose contribution from the low-dose halo is given by the second sum on the right-hand side of Eq. 1. In this case, the sum is taken over HPBs, which, since no sub-PB splitting is applied, coincide in number and position with the physical PBs. Therefore, the weight, *N*_j_, of HPB j is equal to that of the corresponding physical PB. The width of HPB j, σ_HPB,j_, is defined in accordance with Ref. (12) as

(2) $$\sigma_{\text{HPB}}(E,z)=\sqrt{\sigma_{\text{PB}}^{2}(E,z)+\sigma_{\text{LA}}^{2}(E,z_{\text{WE}})}$$

where σ_PB_ is the total width of the contribution from primary protons before the sub-PB splitting, and σ_LA_ is the large-angle component given by the halo dose parameterization. Similar to *u*, and contrary to σ_CPB_ (and thus σ_PB_), σ_LA_ depends only on the initial beam energy and the WEPL.

### Beam Model Parameterizations

Two different parameterizations for the halo fraction, *u*, and the large-angle component, σ_LA_, were investigated. The first parameterization, which will be referred to as the Soukup model, makes use of the unmodified analytical fits to MC data of nuclear interaction products given in Ref. (12), given by

(3) u(E,zWE)=0.052log(1.13+zWE11.2−0.023R0(E))+0.350.0017R02(E)−R0(E)(R0(E)+3)2−zWE2 −1.61×10−9×zWE×(R0(E)+3)2

where, if the right-hand side becomes negative, *u*(*E*, *z*_WE_) is set to 0, and

(4) $$\begin{array}{l} \sigma_{\text{LA}}\left(E,z_{\text{WE}}\right)=2.85+0.0014R_{0}\left(E\right)\times \log\left(z_{\text{WE}}+3\right)+0.06z_{\text{WE}}-7.4\times 10^{-5}\times z_{\text{WE}}^{2}\\ \;-0.22\frac{R_{0}\left(E\right)}{{\left(z_{\text{WE}}-R_{0}\left(E\right)-5\right)}^{2}} \end{array}$$

In the above equations, *R*_0_(*E*) is the Bragg peak (BP) depth in water for a PB of initial energy *E*. Past the BP, both *u* and σ_LA_ are assumed to take the same value as at the BP depth (although this is only stated explicitly for *u* in the original publication). In order to calculate the new values for *E*_S_ and δ for the primary contribution, the radial halo distributions of individual PBs in water were calculated using the expression inside the second sum on the right-hand side of Eq. 1 together with Eqs 3 and 4. The results were then subtracted from the corresponding radial dose distributions obtained from MC simulations to obtain the expected radial dose distribution of the primary particles. For each depth and energy, these were then fitted with a Gaussian function to extract the values for σ_PB_ in water. Since σ_HPB_ in Eq. 1 itself depends on σ_PB_, this process was done iteratively, using σ_PB_ from the single-Gaussian implementation as the starting point. However, due to σ_LA_ generally being at least a factor of two larger than σ_PB_, the exact value of the latter was seen to play a limited role, resulting in the calculation converging after a single iteration. The resulting values of σ_PB_ were finally used to obtain the new values for the parameters *E*_S_ and δ in the same way as for the single-Gaussian beam model (7).

**Figure 1:**
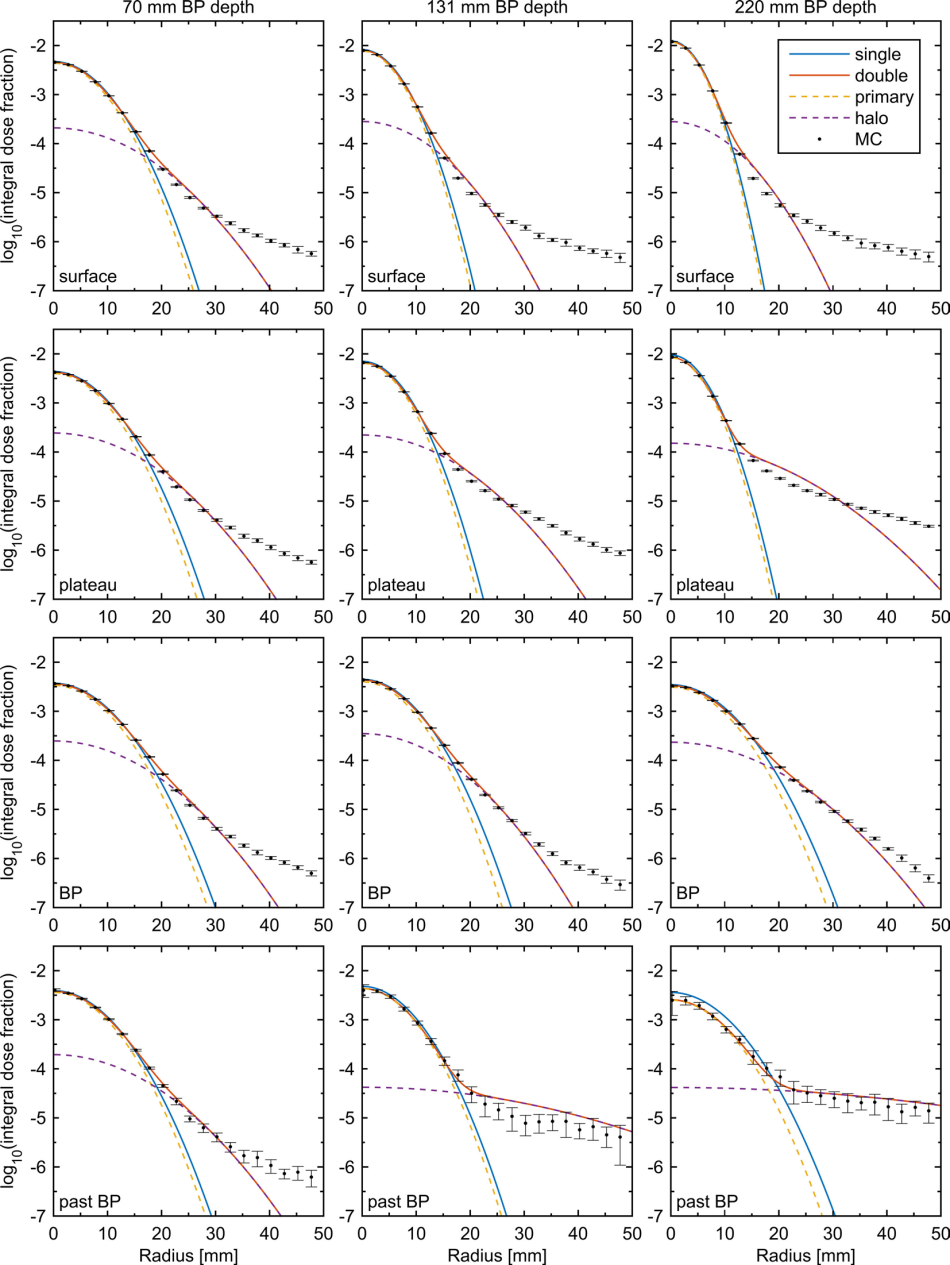
**Radial dose profiles as simulated by MC (black dots with error bars) fitted using single- and double-Gaussian models (solid lines)**. Dashed lines show the components of the double-Gaussian model. The error bars correspond to three times the estimated SD from the MC simulation, and to avoid crowding only every fifth MC data point is shown. Columns, from left to right, correspond to PBs with BP depths 70, 131, and 220 mm. Rows, from top to bottom, correspond to the profiles at the surface, at 40% of the BP depth, at the BP depth, and at 104% of the BP depth. The legend of the top left panel applies to all panels.

**Figure 2:**
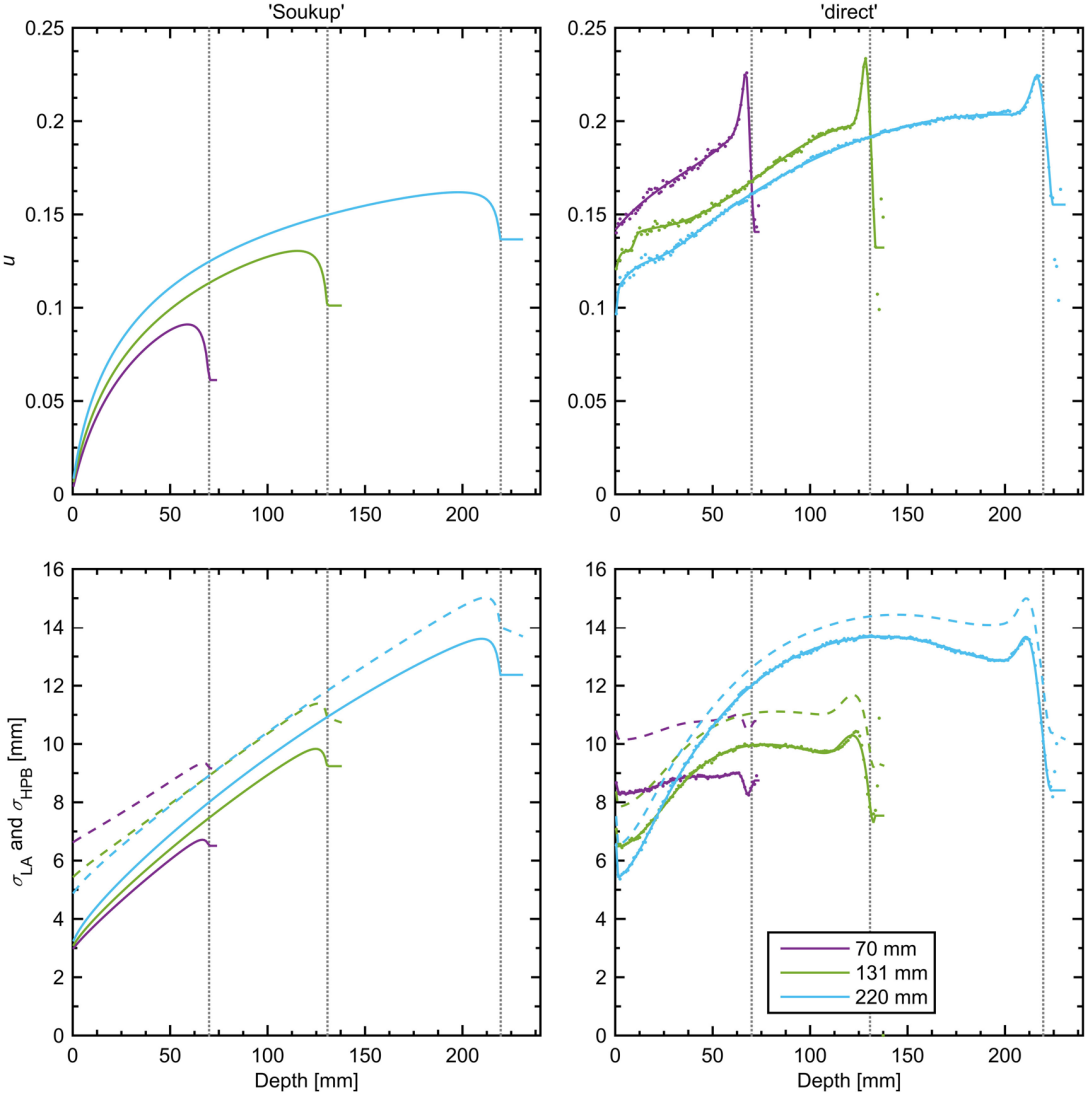
**Halo fraction, *u*, of the total dose (top row), σ_LA_ (bottom row, solid lines), and σ_HPB_ in water (bottom row, dashed lines) for the two parametrizations at three different beam energies**. The individual data points used to fit the splines of the direct model are shown as dots (frequently coinciding with the corresponding line) in the right-hand panels, making visible the oscillating behavior in the model past 102% of the BP depth (with some dots falling outside the panels). Data for each parameterization are shown until 105% of the BP depth, the largest WEPL considered in the calculation. The BP depth for each line color is given by the legend in the bottom right panel and is also indicated by vertical dotted lines.

The second parameterization, which will be referred to as the direct model, relied on fitting sums of two Gaussians directly to the total radial dose distributions obtained from MC simulations, similar to Parodi et al. (17). This was done through a non-linear least squares fit using the trust-region algorithm provided with the Optimization Toolbox of Matlab (Mathworks, Natick, MA, USA). Despite the fact that the radial dose distributions quickly become very small for large radii, the contributions at large radii are important for two reasons. First, the radial distributions do not reflect the larger volumes receiving the contributions from larger radii, which is part of the reason why the low-dose halo is of interest in the first place. Second, since the dose fraction of the halo is expected to be smaller, but the width of its Gaussian larger than for the primary particles, its dose contribution will be dwarfed close to the central axis, and its parameters must thus be determined mainly from the dose at large radii. Therefore, in order for the optimization not to ignore the small contributions at large radii, the contribution to each radial bin was weighted according to the total area of a ring of the same width, i.e., with $\pi \left(r_{\text{i}+1}^{2}-r_{\text{i}}^{2}\right)$ for the bin between radius *r*_i_ and *r*_i+1_. The fit allowed the three parameters σ_PB_, σ_HPB_, and *u* to be obtained simultaneously for each PB energy and depth in water. Values of σ_LA_ for different depths were then obtained from σ_PB_ and σ_HPB_ by rearranging Eq. 2. In order to reduce the noise present in the calculated depth curves for *u* and σ_LA_ (c.f. Figure 2), these were fitted with cubic splines to obtain the final parameterizations. Past the BP, where few charged particles remain in the beam, the basis for using separate Gaussians for charged primaries and secondaries starts to break down. This was characterized by a sharp drop in *u* (likely due to the charged secondaries stopping earlier than primaries), followed by *u* tending toward unity, while σ_LA_ grows very large, consistent with the figures shown in Ref. (17). The behavior is likely explained by the limited number of charged particles past the BP causing the second Gaussian to be fitted to the “aura” of uncharged secondaries (24), which is more appropriately described by a non-Gaussian function (20, 21, 23, 24). Although a double-Gaussian fit still provides some improvement in this region (c.f. bottom row of Figure 1), the aura was ignored, consistent with our approach at shallower depths. Thus, past 102% of the BP depth, the same solution of keeping the values of *u* and σ_LA_ constant as for the Soukup model was employed. The values of *E*_S_ and δ were finally obtained from σ_PB_ in the same way as before.

### Implementation

Incorporating the double-Gaussian beam model in Eq. 1 into the existing PB dose calculation engine could in theory be achieved by carrying out the same calculation procedure twice: once for the primary and once for the halo contributions. However, there are two strong arguments against this solution. First, several parts of the implementation rely on assigning one thread per lateral voxel position. While this works well for the large number of CPBs used to calculate the primary contribution, the number of HPBs is expected to be about nine times lower since no sub-PB splitting is applied. Therefore, small- and medium-sized treatment fields would likely not contain enough HPBs to saturate a modern GPU. Second, several of the intermediates and results obtained in the calculation of the primary contribution, importantly the WEPL and σ_CPB_ (from which σ_PB_ is obtained), are also needed to calculate the halo contribution. Therefore, repeating the whole calculation would either require recalculating the necessary intermediates or keeping large amounts of data in global memory between the two rounds of calculations. Instead, it was deemed more efficient to maintain the structure presented in the original publication [c.f. Figure 2 in Ref. (7)] for the calculation of the primary contribution and to interleave it with the halo dose calculation. While the calculation of primary dose thus remains identical to what was previously presented, the following paragraph describes the changes made to the dose calculation engine in order to accommodate the halo dose calculation.

The only part of the implementation that was significantly modified compared to the original was the one calculating and storing the integral dose and kernel parameter for the CPBs, which was extended to perform the same operations for the HPBs as well. Due to the smaller number of HPBs than CPBs, the additional operations were carried out only by the threads corresponding to CPBs whose positions coincide with that of a HPB. Although it led to the majority of threads being idle during the additional operations, this method was deemed preferable compared to using a separate calculation step for the HPBs, which would in any case not have enough parallelism to saturate the GPU. For each step along the *z*-axis, the widths of the HPBs were calculated according to Eq. 2. The required value of $\sigma_{\text{LA}}^{2}$ was found by linear interpolation into a 2D texture containing the selected parameterization, and $\sigma_{\text{PB}}^{2}$ was calculated by adding in quadrature the PB width at the patient surface, σ_air_ (*E*, *z*_0_), to the value of σ_CPB_ already calculated for the primary contribution. Furthermore, the integral dose for the halo contribution was obtained by multiplying the local IDD contribution, the full PB weight, and *u*, where *u* was again obtained by interpolating into a 2D texture. (A multiplication with (1 − *u*) was similarly introduced in the calculation of the integral dose for the primary contribution.) To be compatible with the efficient KS implementation presented in a previous paper, the obtained values for the integral voxel dose and kernel parameter were then converted to dimensionless voxel units (8). These values were stored in two additional global memory arrays alongside those of the primary contribution. The KS step of the halo dose was identical to that of the primary contribution, and the two KS steps were carried out sequentially for each energy layer. However, due to the different resolutions of the CPB and HPB BEV systems, the BEV halo dose was kept in a separate BEV dose array. For the same reason, the transformation of the BEV halo dose to the global dose grid after completing the calculation for all energy layers also had to be done separately from the BEV primary dose.

### Validation and Benchmarking

The double-Gaussian beam model was validated and benchmarked in the same way as the original dose calculation engine (7). In brief, all the reference dose distributions were obtained using the Fluka MC code, employing the beam line parameters and nozzle geometry of the Centro Nazionale di Adroterapia Oncologica (CNAO) treatment center in Pavia, Italy (25, 26). For the single PB validation, the radial dose distribution from a PB of BP depth 220 mm in water was calculated using 1 mm CPB spacing and a global dose grid resolution of 1 mm × 1 mm × 1 mm and was compared with the corresponding reference PB. For patient case validation, a base-of-skull plan for a 55.6 cm^3^ planning tumor volume target consisting of two oblique fields of 38 and 45 energy layers was used. The PB spacing (and thus HPB spacing) was 3 mm, the CPB spacing was again set to 1 mm, and, in order to match the resolution of the provided reference MC simulation, a global dose resolution of 2 mm × 2 mm × 2 mm was used. The same patient case was also used in the benchmarking. In addition, benchmarking was carried out on a plan for a cubic target of side length 100 mm extending 100–200 mm below the surface of a water tank and consisting on 20 energy layers. For this plan, the PB spacing was 3 mm, the CPB spacing was set to 1 mm, and a global dose resolution of 1 mm × 1 mm × 1 mm was used. The calculations were carried out on a Tesla K40 GPU from Nvidia (Santa Clara, CA, USA) with 2880 cores running at a clock frequency of 875 MHz.

## Results

### Beam Model Parameterizations

The results of directly fitting a sum of two Gaussians to the radial profiles of PBs of three different energies in water are shown in Figure 1. Each PB is shown at four depths, corresponding to the surface, 40% of the BP depth, the full BP depth, and 104% of the BP depth, and for comparison direct fits using a single-Gaussian are shown. A clear deviation from a single Gaussian is seen for all beam energies and at all depths, including the surface, indicating that nuclear interactions may not be the only factor contributing to the low-dose halo in the beam line modeled. For larger radii, it is clear that even an ideal double-Gaussian model breaks down far away from the central axis. However, for all depths up until just after the BP, this happens at a dose level, which is at least one order of magnitude smaller than for the single-Gaussian model.

Figure 2 shows curves of *u* and σ_LA_ according to the two parameterizations considered and for three beam energies with BPs at 70, 131, and 220 mm depth in water. A striking feature of this figure is the large difference in the shapes and magnitudes of both *u* and σ_LA_ between the models, especially at low beam energies. This shows that the assumptions made in the parameterization do indeed impact the resulting beam model. Although it was hard to perform a quantitative comparison with the data shown by Parodi et al. (17), the shapes and magnitudes of their curves for the CNAO treatment center seem to agree with those of the direct model presented here, as expected from the similar method used. The direct model also shows the closest agreement with the measurement-based parameterization by Pedroni et al. in terms of the shapes of the curves for *u* and σ_HPB_, although their values of *u* were seen to be almost half the size, and their values of σ_HPB_ a few millimeters larger (11). In addition to the differences in *u* and σ_LA_, a difference was also seen in the new values of *E*_S_, which were 13.8 MeV for the Soukup model and 13.0 MeV for the direct model. The new values of the empirical correction δ were, in the same order, 0.00 and 0.06 mm. The low values are expected since any major deviations from the multiple-scattering model should now be incorporated in the halo contribution. For the Soukup model, the halo fraction is close to 0 at the surface, which means that the width of the primary contribution at the surface should be given by the total PB width in air, as was the case for the single-Gaussian beam model. However, as can be seen from Figure 2, the halo fraction at the surface is non-zero for the direct model. The calculated PB width in air at the surface thus corresponds to the effective width of the primary and halo contributions taken together, and subsequently the width of the primary contribution must be smaller than this. It was seen that, in order to obtain the correct total beam width at the entrance point, the width of the primary contribution had to be set 2–4% smaller than the calculated PB width in air across the different energies. Therefore, when using the direct model, the entry width used in the calculation of the weights for the primary CPBs was set to 97% of the width calculated in air.

### Validation

**Figure 3:**
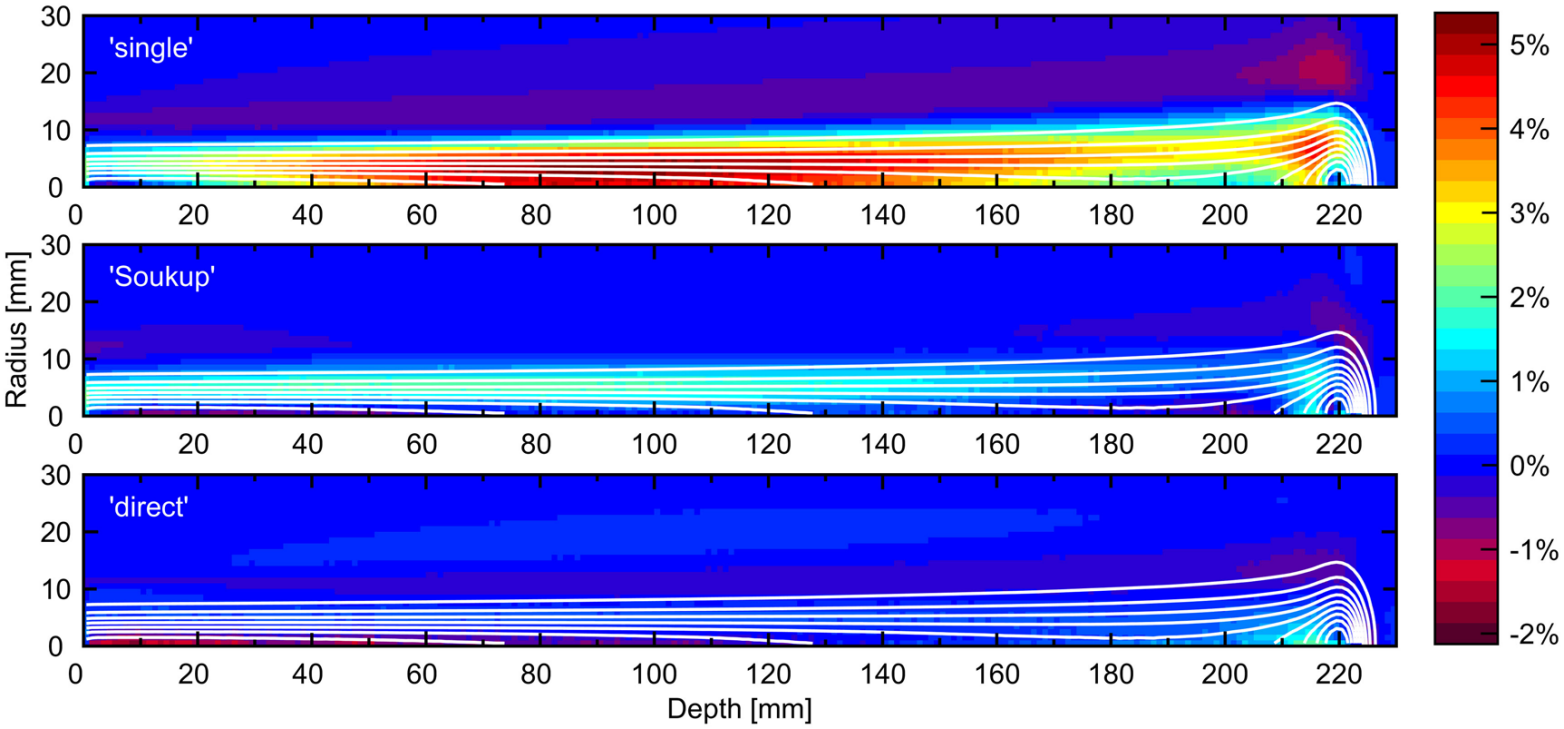
**Errors in radial distributions as a percentage of maximum dose for a PB of 220 mm BP depth when comparing the presented implementation to a MC simulation**. The panels show the previously presented single-Gaussian implementation (top), the Soukup model (middle), and the direct model (bottom). The contours show the MC isodose curves, with each line corresponding to a multiple of 10% of the max dose.

Figure 3 shows the difference in calculated dose for a PB of 220 mm BP depth when comparing the presented dose calculation engine using different halo dose parameterizations with the reference MC simulation. The result obtained for the previously presented single-Gaussian beam model is included for reference, showing that both parameterizations considerably reduce the average error in comparison. Unsurprisingly, the smallest average error was achieved for the direct model, with the Soukup model performing surprisingly well despite the lack of beam line-specific tuning. The error ranges in Figure 3 were −0.8 to 2.1 and −1.8 to 2.0%, respectively, for the Soukup and direct models, compared to −1.1 to 5.3% for the single-Gaussian model. The small value of the lower boundary of the direct model was caused by the large underestimation seen along the central axis close to the surface in Figure 3.

**Figure 4:**
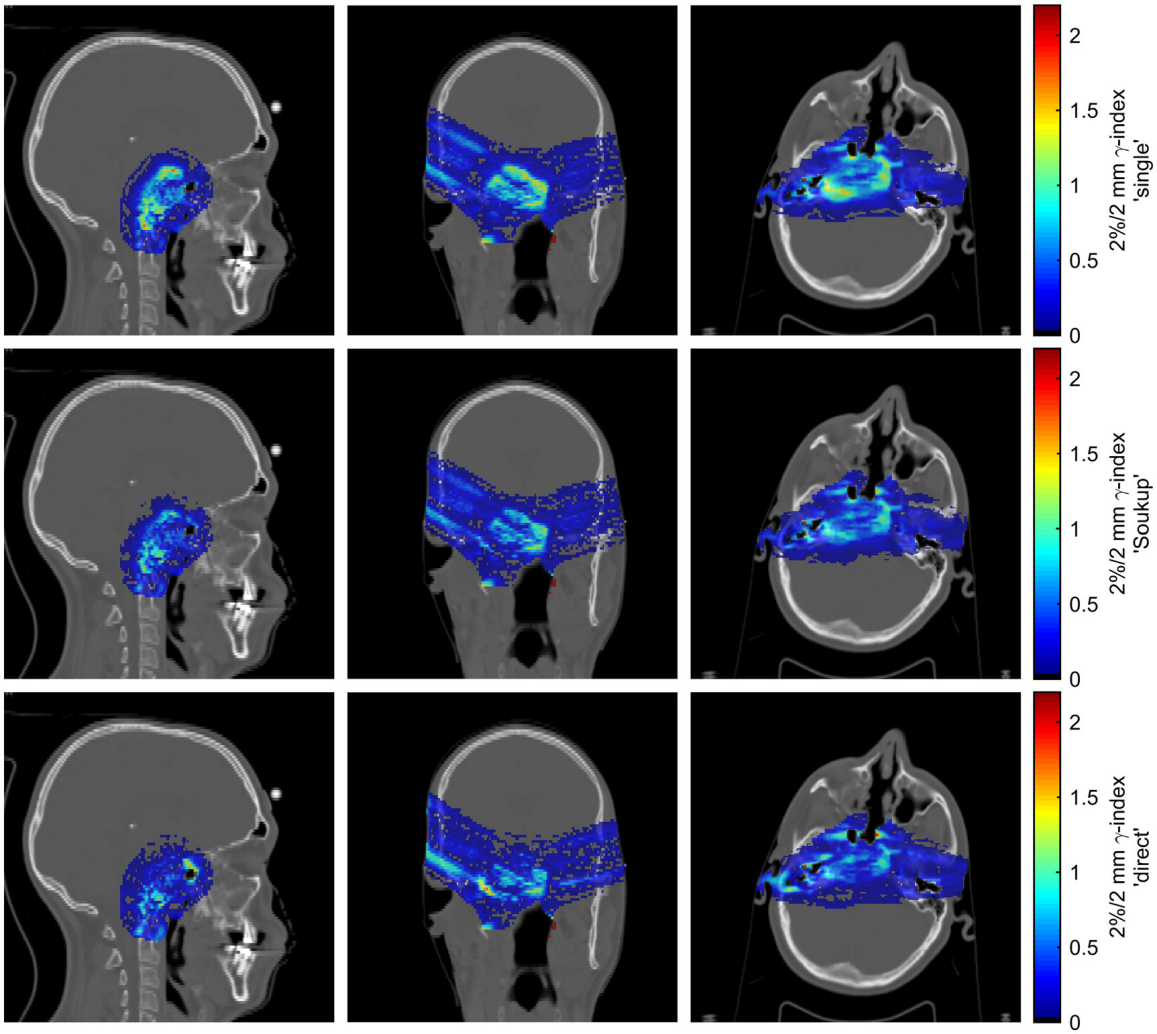
**Maps of the 2%/2 mm γ-index for the patient case for different beam model parametrizations**. The rows correspond, from top to bottom, to the single-Gaussian, Soukup, and direct models. Columns, from left to right, show sagittal, coronal, and axial slices roughly through the center of the target.

Figure 4 shows γ-index maps according to the 2%/2 mm criterion for the reference patient case. Although the γ-index is a poor measure of the agreement in the low-dose region, better modeling of the halo dose is expected to be somewhat reflected in the γ-index due to the contribution of overlapping halos from multiple PBs to the high- and medium-dose regions. The γ-index passing rates for voxels receiving at least 10% of the prescription dose according to the 2%/2 mm criterion were 97.9% for the Soukup model and 97.4% for the direct model, compared to 96.7% for the single-Gaussian model. For the less strict 3%/3 mm criterion, the passing rates for the Soukup and direct models were 99.4 and 99.2%, respectively, similar to the 99.2% obtained for the single-Gaussian model.

### Benchmarking

Despite the differences seen in Figure 2, the performance of the two parameterizations of the double-Gaussian beam model was very similar. The calculation times for the patient case were 241 and 244 ms, respectively, for the Soukup and direct models. This constitutes increases in the calculation time of 8 and 9% compared to the 224 ms required by the single-Gaussian model. The increase in calculation time for individual energy layer was seen to be larger and shifted toward smaller energy layers: the shortest calculation time (excluding memory transfers and deallocations) for an energy layer was 3.2 and 3.3 ms for the two models, or about 50% longer than for the single-Gaussian model, whereas the longest calculation time was 8.1 and 8.4 ms, or around 25% longer than for the single-Gaussian beam model. The overall increase in calculation time was slightly larger for the deeper test case consisting of a cubic target in water, which required 153 ms using the Soukup model and 157 ms using the direct model, which is 13 and 16% longer than the 135 ms required by the single-Gaussian model. The calculation times for the shallowest energy layer were 7.4 and 7.6 ms, and for the deepest energy layer 16.5 and 16.8 ms. Compared to the 6.0 and 13.3 ms required by the single-Gaussian beam model for the shallowest and deepest energy layer, this corresponds to an increase of roughly 25% in both cases.

## Discussion

### Validity of Approach

The reason for including two parameterizations of the double-Gaussian beam model was primarily to investigate their effect on the calculation time. Consequently, the tuning of the beam models implemented was kept as simple as possible, without much of the time-consuming and detailed analysis that is associated with the commissioning of a clinical dose calculation engine. Therefore, the result obtained using the presented models may not be representative of the selected algorithm or the parameterization methods themselves, other than to serve as a lower bound for their accuracy. On the contrary, smaller errors can be expected for both models if, for example, model-specific tuning or energy-dependent tuning were used, as discussed in the following subsection. The two models are, however, expected to capture the essence of the two main types of parameterizations, namely, those aimed specifically at modeling the contributions from nuclear interactions and those based on direct fitting of lateral profiles (which thus also include other contributions to the halo). Therefore, the presented parameterizations should be indicative of how sensitive the performance of the implementation is to the model used.

It should be pointed out that in the original implementation (12), the dose from CPBs and HPBs was calculated directly in the global dose grid. Therefore, the lack of sub-PB splitting for the halo dose served only to limit the number of HPBs but did not reduce the resolution used in the KS step. However, using a single HPB per PB already limits the effective resolution of the halo calculation. Therefore, when the KS is carried out in BEV coordinates as here, using the same reduced resolution also in the KS step should not affect the accuracy of the calculation, provided that the kernel varies slowly across the BEV voxels. Since, compared to the CPBs, the HPB kernel widths are increased by a similar amount to the voxel spacing, this should hold also for the HPBs. Thus, the effect of the lower resolution in the KS step is not expected to affect the accuracy of the calculation noticeably.

### Beam Model Parameterizations

Since the Soukup model was implemented directly from the analytical expressions given in Eqs 3 and 4, little room was left for adjustments of the parameterization itself. Despite this, it resulted in a clear improvement over the single-Gaussian model both in the dose for a single PB in Figure 3 and in the 2%/2 mm γ-index passing rates in the patient case. The overall improvement in the γ-indices compared to the single-Gaussian beam model can also be seen in Figure 4. Still, the model assumes that there is no halo dose at the surface (c.f. Figure 2), whereas the MC results shown in Figure 1 clearly suggest the presence of such a contribution across the therapeutic range of energies for the beam line considered in this work. Therefore, using a more accurate description of the beam profile in air, such as a sum of two Gaussians, would likely further reduce the errors in the plateau region. A simple version of such an improvement would affect only how the weights are distributed between CPBs, and thus would have a negligible effect on the calculation time.

Although the direct parameterization method showed the best overall agreement for the single PB in water in Figure 3, the agreement was slightly worse than for the ideal fits of a sum of two Gaussians seen in Figure 1. The main reason for this is thought to be that, in order to more accurately model the multiple scattering in beams passing through different materials, the CPB widths of the primary contribution were calculated, as previously described (7), rather than obtained directly from the fit in water. Therefore, the width of the primary contribution at any given depth is constrained by the parameters *E*_S_ and δ, and, contrary to the halo contribution, cannot vary arbitrarily. More interestingly, the better average agreement did not translate to the γ-index passing rates, where the Soukup model showed better results. Looking at Figure 4, the γ-indices for these two models display similar behavior except for in limited regions where the indices for the direct model are considerably higher (c.f. the lower part of the left field in the coronal view of Figure 4). The reasons for this are not entirely clear. One explanation could be the relatively large underestimation of the PB central axis dose to a small number of voxels close to the surface seen in Figure 3. Another could be the larger halo fraction, which excludes more than just the nuclear interaction products from the more accurate physical modeling of the primary contribution. A third might be the rather arbitrary reduction of the width of the PB entrance profile that was employed to make the direct parameterization compatible with the existing beam model in air. In an improvement of the direct parameterization, constraints set on the primary contribution by *E*_S_ and δ could thus be included already in the fitting of the sum of two Gaussians. Furthermore, since the fit was seen to be relatively flexible, a preference to limit the size of the halo fraction could be included in order not to remove too much of the weight from the primary contribution. Finally, the empirical shrinking of the entrance dose applied here could be more accurately incorporated in the description of the beam profile in air.

### Calculation Times

The benchmarking showed that, using one HPB per physical PB, the incorporation of a double-Gaussian beam model into the presented dose calculation engine lead to an increase in the total calculation time of no more than 16% for the two treatment plans tested. The increase in calculation time was larger for individual energy layers, ranging from about 50% for a small, shallow energy layer to around 25% for energy layers large enough to saturate the GPU. Both in the case of complete treatment plans and single energy layers, the increase in calculation time varied by only a few percentage points between the two parameterization models tested. These findings have two major consequences. The first is that employing either of the investigated parameterizations of the double-Gaussian beam model does not impact upon the suitability of the presented dose calculation engine for use in online dose calculation applications; calculation times of 16.5–16.8 ms for the deepest energy layer of the presented plans are still considerably shorter than the time between energy layers or the duration of a typical motion phase. The second is that as long as the presented implementation of the double-Gaussian beam model is used, the calculation time is unlikely to change significantly for other, beam line-specific or more sophisticated, parameterizations of the halo dose. Together, these indicate that, using a single GPU, it is possible to achieve fast enough calculation times for online dose calculation while maintaining the same accuracy as a widely adopted clinical algorithm, independent of the specific beam line.

The larger increase in calculation time for single energy layers than for complete treatment plans can be explained by the varying fractions of the total calculation time spent on different steps of the calculation. The calculation of complete treatment plans was dominated by the KS step, which, using the single-Gaussian beam model, was responsible for 76% of the calculation time for the patient plan and 88% of the calculation time for the cubic test case (excluding the time spent on memory transfers in both cases). Using the double-Gaussian parameterizations, the increase in calculation time for the KS step was 3–5% for the patient case and 14–18% for the cubic test case, which therefore resulted in increases of a similar order in the total calculation time for entire plans. For the individual energy layers, on the other hand, where the KS step is carried out only once, the calculation times of the steps that are carried out once per beam direction become comparable to the KS time. The calculation times for these steps generally increased more than that for the KS step when going from the single-Gaussian to the double-Gaussian beam model. In particular, the time required to set up the calculation and allocate memory for BEV intermediates and the time to copy the dose distribution to texture memory both increased by between 20 and 40%. Furthermore, due to the larger number of voxels reached by the halo, the time required to transform the dose from the BEV coordinate system to the global dose grid roughly doubled. In the light of this, the overall increases in calculation time of 50% for a small energy layer or 25% for large energy layers are not surprising.

## Conclusion

We have described how a double-Gaussian beam model was incorporated into an existing implementation of the PB algorithm running on a GPU while avoiding the prohibitive increase in calculation time expected from the large halo width. The increase in calculation time was not larger than 16% for entire treatment plans, and about 25% for large energy layers, which are the most time-consuming to calculate. Therefore, the addition of a double-Gaussian beam model does not alter the suitability of the presented implementation for use in online dose calculation. The calculation time was further shown to be relatively unaffected by the specific parameterization used to describe the halo dose contribution. Despite the calculation of the halo contribution being simplified compared to that of the primary, it was based on the same algorithm as a widely used commercial TPS. Therefore, it is expected that with adequate tuning, it will be able to reproduce with sufficient accuracy the halo dose of a general beam line. Based on these observations, we conclude that, using a single GPU, dose distributions from individual energy layers can be calculated with comparable accuracy to a modern clinical TPS well within the time of a typical motion phase or change of beam energy.

## Author Contributions

JS, RA, and RJ contributed to the conception and design of the study, and JS was responsible for the acquisition and analysis of the data. JS drafted the work that was critically revised for important intellectual content by RA and RJ. All authors approve of the submitted version and agree to be accountable for all aspects of the work.

## Conflict of Interest Statement

The authors declare that the research was conducted in the absence of any commercial or financial relationships that could be construed as a potential conflict of interest.
